# A Study on the Evaluation of Thermal Insulation Performance of Cellulose-Based Silica Aerogel Composite Building Materials

**DOI:** 10.3390/polym16131848

**Published:** 2024-06-28

**Authors:** Jeo Hwang, Yoonmi Kim, Jooyoung Park, Dongho Rie

**Affiliations:** 1 Graduate School of Safety Engineering, Incheon National University, Incheon 22012, Republic of Korea; hjojjang@inu.ac.kr; 2 Department of Safety Engineering, Incheon National University, Incheon 22012, Republic of Korea; 1231yunmi@naver.com (Y.K.); wndud3539@naver.com (J.P.); 3 Fire Disaster Prevention Research Center, Incheon National University, Incheon 22012, Republic of Korea

**Keywords:** cellulose, thermal conductivity, thermal diffusivity, building materials

## Abstract

Buildings utilize both inorganic and organic insulation materials to conserve energy and prevent heat loss. However, while exhibiting excellent thermal insulation performance, organic insulation materials increase the risk of fire due to the emission of intense heat and toxic smoke in the event of a fire. Conversely, inorganic insulation materials are characterized by a lower thermal insulation performance, leading to an increase in the weight of the building with extensive use. Therefore, the necessity for research into new insulation materials that address the drawbacks of existing ones, including reducing weight, enhancing fire resistance, and improving thermal insulation performance, has been recognized. This study focuses on evaluating the enhancement of the thermal insulation performance using novel building materials compared to conventional ones. The research methodology involved the incorporation of porous aerogel powders into paper-based cellulose insulation to improve its insulating properties. Samples were prepared in standard 100 × 100 mm^2^ panel forms. Two control groups were utilized: a pure control group, where specimens were fabricated using 100% recycled cardboard for packaging, and a mixed control group, where specimens were produced using a mixture ratio of 30 wt% ceramic binder and 40 wt% expandable graphite. Experimental group specimens were prepared by increasing the aerogel content from 200 to 1000 mL under each condition of the control groups (pure and mixed) after mixing. The thermal insulation performance of the specimens was evaluated in terms of thermal conductivity and thermal diffusivity according to ISO 22007-2 (for solids, paste, and powders). Through this study, it was found that the thermal insulation performances of the pure control and experimental groups improved by 16.66%, while the mixed control and experimental groups demonstrated a 17.06% enhancement in thermal insulation performance with the addition of aerogel.

## 1. Introduction

The recent increase in greenhouse gas emissions has led to a rapid escalation of global warming. Surface temperatures on Earth have shown a swift rise since 1970, resulting in significant climate change. In response, the international community has adopted low-carbon policies to minimize greenhouse gas emissions. Enhanced thermal insulation in buildings is anticipated to contribute to a reduction in the use of fossil fuels, thus making it a key objective of low-carbon policies.

Furthermore, the global spread of COVID-19 has significantly shifted towards non-face-to-face lifestyles, resulting in a sharp increase in parcel delivery services [1]. In 2020, the number of parcels delivered reached 3.37 billion, marking a 20.9% increase from 2.798 billion in 2019. Recycling paper, commonly disposed of after use, can save 17 trees per ton (907 kg). Research is underway to utilize cellulose, a component of paper, for producing construction materials, aiming to achieve both low-carbon emission policies and recycling objectives.

The main components of paper are cellulose, hemicellulose, lignin, and various additives. Cellulose constitutes about 40% to 60% of paper and serves as the primary structural element, providing strength and durability. Hemicellulose accounts for approximately 10% to 30% and consists of shorter fibers compared to cellulose. Lignin makes up about 15% to 30% and binds cellulose and hemicellulose together, enhancing their strength [2].

**Figure 1:**
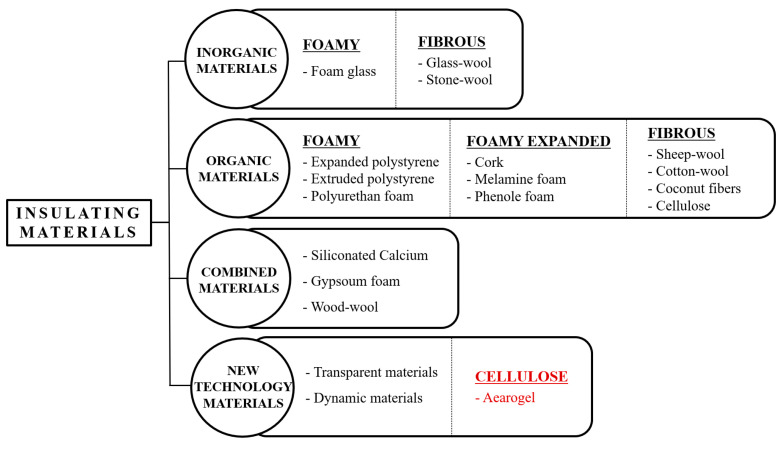
Classification of existing insulation materials and the material mix for this experiment.

Traditional insulation materials used in buildings primarily aim to prevent energy loss. Depending on the materials used, traditional insulation can be classified as shown in Figure 1.

According to a study by Han-Hsi, L., et al., the toxicity indices of organic foaming materials and polyurethane, as well as polyethylene-based building materials, evaluated using the NES-713 assessment method, were found to be higher than 10, indicating a high risk of smoke toxicity [3]. Despite their efficient energy storage capabilities, organic insulation materials are susceptible to fire hazards. Consequently, in the event of a building fire, the expansion of the fire and the emission of toxic gasses can result in significant casualties.

As awareness of the shortcomings of organic insulation materials grows, there has been an increasing use of inorganic insulation materials. However, inorganic insulation materials are characterized by a lower insulation efficiency compared to organic ones and pose challenges in installation. Hence, research on new technology materials with excellent insulation efficiency is in demand.

Aerogel is a lightweight and porous insulating material characterized by its nanoporous structure. Aerogels are composed primarily of silica, with oxygen arranged in a dual-structure, double-bonded form on both sides. Silica finds application in various fields such as nanocomposites, construction, and chemical industries. Historically, the high production cost of aerogel has hindered its widespread application as a conventional material. However, recent advancements in mass production technology have lowered production costs, leading to its utilization in both research and consumer goods.

In 1931, Steven Kistler introduced aerogel, which is lightweight and porous. Silica, a basic component of soil and rocks, including granite and basalt, is the most abundant mineral on Earth and serves as the fundamental material for aerogels [4]. Silica finds application in various fields such as nanocomposites, construction, and chemical mechanical polishing [5]. Research on silica-based aerogel manufacturing methods for commercialization is ongoing [6]. Silica aerogel exhibits outstanding insulation properties due to its ability to transmit only about 1/100th of the heat compared to ordinary glass [7]. With pore sizes ranging from 5 nm to 70 nm, silica aerogel contains a variety of cells filled with air, which block heat transfer and enhance insulation performance [8]. The thermal conductivity of silica aerogel, a porous material, is approximately 14 mW/m·K [9].

Initially, aerogel was mainly used in advanced chemical products for aerospace and chemical industries, with limited application in construction materials due to high production costs [10]. However, in the early 2000s, aerogel materials began to be used in buildings, primarily as aerogel blankets [11]. Aerogel-based construction materials, such as aerogel blankets (thermal conductivity around 15 mW/m·K) and aerogel boards (thermal conductivity around 16~19 mW/m·K), are utilized in buildings [12]. In addition to insulation performance, the sustainability of construction materials is crucial for increasing the lifespans of buildings [13].

Sambucci et al.’s study identifies the emerging use of fiber-reinforced aerogel blankets (FRABs) as an alternative insulation material for cryogenic tanks used in liquefied natural gas (LNG) transportation. They found that compared to the traditionally used back-filled perlite-based system for transporting liquefied natural gas (LNG), it allows for a thinner outer shell, stores more material, and reduces the weight of LNG transportation semitrailers [14].

Liu et al. studied the thermal conductivity of specimens as the mass fraction of expanded graphite increased from 2 wt% to 10 wt%. The measured results indicated a linear increase in thermal conductivity. Specifically, the measured value increased from 0.2055 to 0.5218 W/m·K. The thermal conductivity of the mixture with 10 wt% expanded graphite added was 3.28 times higher than before the addition of expanded graphite. The increase in thermal conductivity was found to correspond to a decrease in thermal insulation performance [15].

This study produced specimens by pulping paper to extract its main component, cellulose, and adjusting the content of porous aerogel. Specimens mixed with paper and porous aerogel were classified as the pure test group. The composite test group was also created by combining porous aerogel with 30 wt% ceramic binder and 40 wt% expanded graphite to delay fire spread [16]. The thermal insulation performance of the fabricated specimens was evaluated using the ISO 22007-2 method, through which their thermal conductivity and thermal diffusivity were measured.

## 2. Materials and Methods

### 2.1. Materials

The powdered silica aerogel used in this study was manufactured by Hongda Technologies. Table 1 shows the physical characteristics of the powdered silica aerogel provided by the manufacturer.

The paper was purchased from a local recycling center in Incheon, South Korea, and was used as the primary raw material for this research. The paper used in this study was cardboard. The cardboard was recycled from paper previously used for delivery boxes. The ceramic binder used in this experiment was supplied by Seonjin Chemicals Co., Ltd., In-cheon, South Korea, under the product name ‘FP-100’. The ceramic binder comprised 48% MgO, 17% SiO_2_, 14.6% Al_2_O_3_, and other components.

Expandable graphite, obtained from Samjeong C&E in Gyeongsan, Hwa-seong, South Korea, was used in the form of a 100-mesh product. Expandable graphite is characterized by its porous structure, which expands several hundred times upon heating, leading to a layer separation phenomenon and making it suitable for use as a material for flame retardancy [17]. The addition of expandable graphite in the composite material ultimately reduces the HRR and THR values of the experimental specimens [18]. Therefore, in this study, expandable graphite and ceramic binder were used as additive materials to investigate the insulation effect of aerogel under mixed conditions.

### 2.2. Aerogel Cellulose Specimen Manufacturing Method

**Figure 2:**
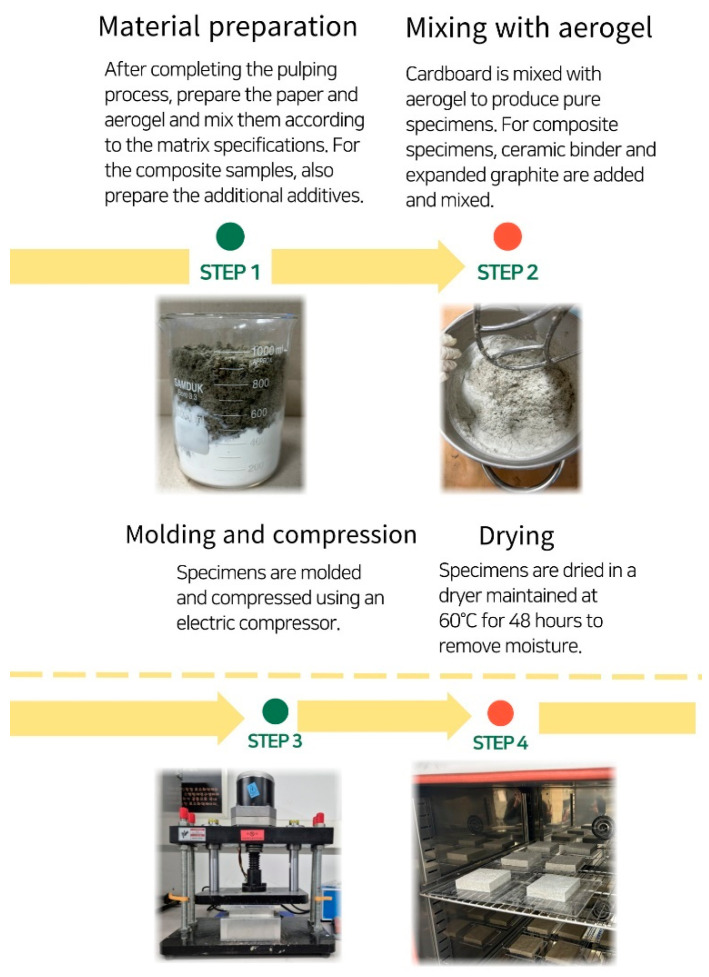
The overall fabrication method of the specimens.

The method of specimen fabrication proceeded in four main steps. In the first step, cardboard was pulped into fine particles. In the second step, pure specimens were fabricated by mixing with aerogel. In this step, ceramic binder and expanded graphite were added and mixed for composite specimens. In the third step, specimens were molded using a molding machine and compressed with an electric compressor. In the fourth step, they were dried in a dryer maintained at 60 °C for 48 h to remove moisture. Specimens molded over four steps were then subjected to stability evaluation under conditions of 23 (±2) °C temperature and 50 (±5)% humidity for 2 days in a constant temperature and humidity chamber. Three specimens of each condition were prepared for all samples, each measuring 10 cm × 10 cm in size. Figure 2 shows the specimen fabrication process.

**Figure 3:**
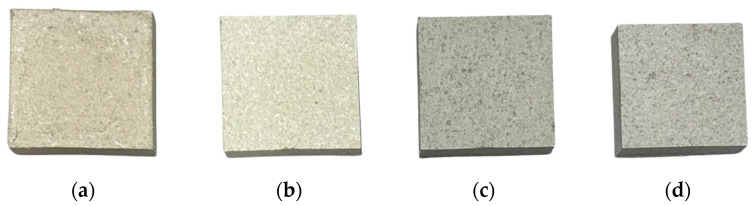
The shapes of mixed specimens of paper and aerogel: (**a**) A-400, (**b**) A-600, (**c**) A-800, (**d**) A-1000.

To evaluate the insulation performance of cellulose and aerogel mixed specimens, pure control specimens devoid of any additives were prepared. As the experimental groups, specimens with increased aerogel content were fabricated from the pure control specimens. Table 2 lists the compositions of the cellulose and aerogel mixed specimens. During specimen fabrication, organic compounds were used to induce bonding between cellulose and aerogel. In this study, the paper was measured in grams (g) for the blending ratio, while the ceramic binder and expandable graphite were adjusted in proportion to the weight of the paper, maintaining the wt% unit used previously. As aerogel is inherently lightweight, volume, measured in cubic centimeters (mL), was utilized instead of weight. Similarly, organic compounds were measured in grams (g) to compose the specimen materials, consistent with the weight-based measurement of paper. In this study, a substance based on a certain amount of ethanol as an organic compound was used. Figure 3 shows the appearances of the specimens in the pure control group.

**Figure 4:**
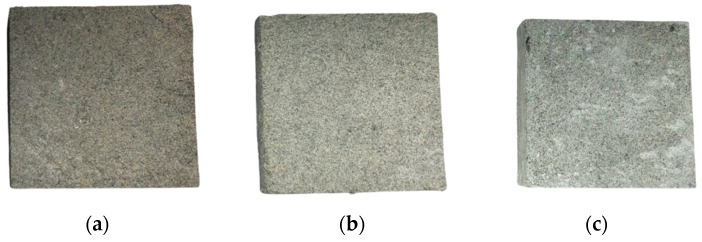
Appearances of the fabricated specimens (**a**) with Aerogel 200 mL, (**b**) with Aerogel 600 mL, (**c**) with Aerogel 1000 mL.

The composite control specimens were fabricated under conditions synthesized with a ceramic binder and an expandable graphite ratio of 30 wt% and 40 wt%, respectively. Composite experimental specimens were prepared by increasing the aerogel content from the composite control specimens’ composition ratio. In the composite experimental specimens, the aerogel, used as a variable, was applied in quantities of 200 mL, 600 mL, and 1000 mL. The same method and unit of material mixing used for the pure control and experimental specimens were also applied to the composite control and experimental specimens. Table 3 lists the mixing ratios and components of the composite experimental specimens. Figure 4 shows the appearances of the composite experimental specimens.

### 2.3. Evaluation of Insulation Performance (Description of ISO 22007-2 Test)

The ISO 22007-2 test method typically measures thermal conductivity (λ) values within the range of 0.01 W/m⋅K λ < 500 W/m⋅K. Additionally, the value of thermal diffusivity (α) is measured within the range of 5 × 10^−8^ m^2^/s < α < 10^−4^ m^2^/s. The measurement method calculates the measured values according to the ISO 22007-2 standard by applying heat to the specimen and observing temperature changes [19].

The thermal performance measurement instrument used was the “MP-V” product manufactured by “Thermtest”, Canada. The measurement method of the MP-V product adheres to the “ISO 22007-2 test” method. For solids, the measurement was conducted using the Transient Plane Source (TPS) sensor. The TPS sensor, designed for solid measurements, consists of a double spiral of encapsulated nickel between insulation layers. The measurement range of thermal conductivity with this sensor is wide, from 0.005 to 1800 W/m·K. The measurement procedure involves placing the specimen between the two sides of the sensor, fixing it in place, and then measuring thermal conductivity and thermal diffusivity after heating the equipment. Furthermore, for each set of three specimens prepared under the same conditions, measurements were taken at two random points per specimen. Consequently, a total of six measurements were conducted per specimen for the thermal conductivity evaluation.

## 3. Evaluation Results

### 3.1. Physical Properties of the Specimens

Table 4 presents the physical characteristics of the specimens fabricated for the experimental group. The average initial dry weight of the pure experimental group specimens was determined to be 199.8 g. After the drying process, the average weight was found to be 88.5 g. Aerogel possesses the inherent property of hydrophobicity [20], leading to induced mixing using a low concentration of organic compounds. The decrease in weight after drying confirmed the evaporation of water and organic compounds present during the manufacturing process. The moisture content evaporated during the drying process was observed to be an average of 55.7% in the pure experimental group specimens. Additionally, in the composite control group specimens, a moisture content of 58.2% was observed. Equation (1) represents the calculation method for moisture content in the experiment:

MC [%] = [(Wm − Wd)/Wm] × 100, (1)

where the variables have the following meanings: 

MC = Moisture content [%];

Wm = Weight of sample before dry;

Wd = Weight of sample after dry.

The area and height were measured using a specimen after drying. The molding size was constant in the molding process, and the height was measured based on the height after drying. In addition, the weight after drying was measured. Therefore, the method of calculating the density used the following formula.

(2) $$Density(\frac{g}{{cm}^{3}})=\frac{weight\;after\;drying(g)}{Volume\;after\;drying({cm}^{3})}$$

The density of the pure experimental group specimens averaged 0.41 g/cm^3^. This value remained consistent in the composite experimental group specimens, also measuring at 0.42 g/cm^3^. Panyakaew et al. conducted research utilizing coconut husks and sugarcane to produce insulation materials, achieving a density of 0.35 g/cm^3^ [21], while Aliaksandr Bakatovich et al. achieved densities ranging from 0.2 to 0.25 g/cm^3^ using straw-based cereals [22]. Furthermore, according to Ahn et al., specimens made from cellulose-based paper had a density of 0.25 g/cm^3^ [23]. The specimens produced in this study exhibited higher densities compared to conventional insulation materials cited in previous research.

### 3.2. Structural Characteristics of the Specimens

**Figure 5:**
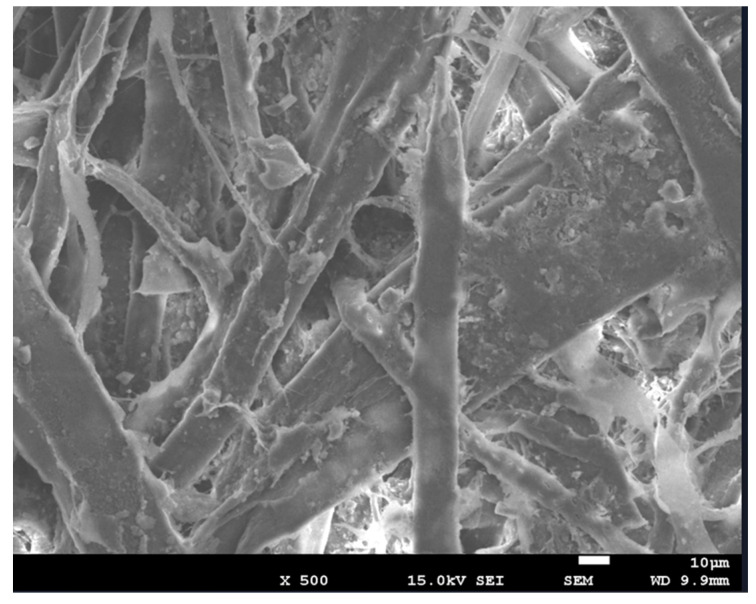
The appearance of the control specimen made entirely of paper.

The structural characteristics of the specimens were measured using “FE-SEM_7001F”. The specimens used for the measurement were control specimens composed solely of paper. For the control specimens, both a control sample containing 1000 mg of porous aerogel and an experimental sample were used. By comparing the structural differences between the control and experimental specimens, the synthesis of aerogel was evaluated. Figure 5 shows the appearance of the control specimen.

The control specimens exhibited materials presumed to be binders used in conventional cardboard production, along with cellulose, lignin, and other substances. These specimens showed a solid structure with fibers intertwined, suggesting the absence of porous materials.

**Figure 6:**
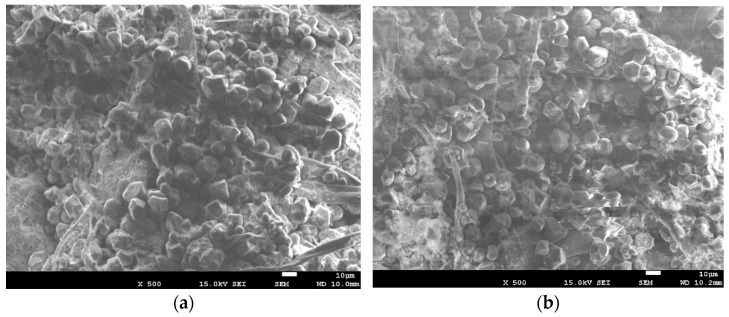
Appearances of the control specimens composed solely of paper. (**a**) Pure test group specimen. (**b**) Composite test group specimen.

In contrast, the control specimens revealed the presence of porous aerogel. Figure 6 shows SEM images of the experimental specimens. In contrast to the control group, a spherical porous aerogel structure was observed, indicating its adsorption in the vicinity of cellulose in a spherical form.

Additionally, quantitative EDS analysis was conducted on the SEM images of the experimental specimens. Table 5 presents the EDS analysis results of the experimental specimens. The analysis confirmed the presence of aerogel, based on silicon (Si) and oxygen(O).

### 3.3. Results of Insulation Performance Evaluation

**Figure 7:**
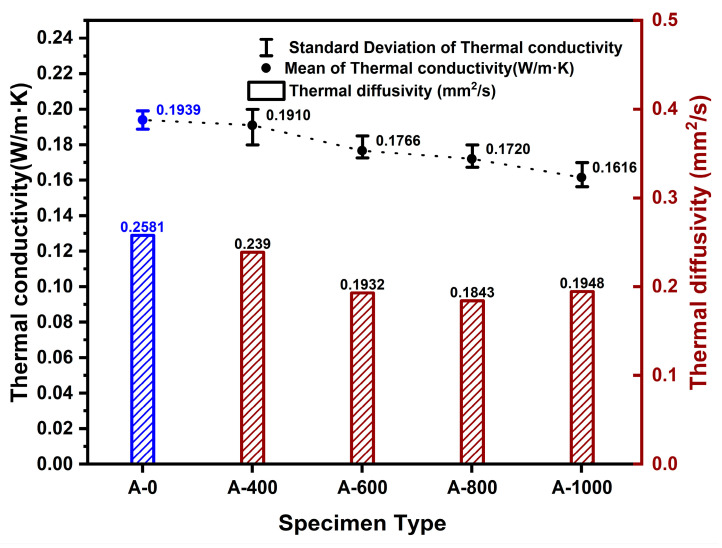
Changes in thermal conductivity and thermal diffusivity according to aerogel content. (Blue: composite control group, Red: composite experimental group).

The thermal conductivity of the pure control group specimens, without aerogel, was determined to be 0.1939 W/m·K. For the pure experimental group specimens composed of aerogel and paper, the thermal conductivity ranged from 0.1901 to 0.1616 W/m·K. Figure 7 shows the measured thermal conductivity and thermal diffusivity values for the pure control and pure experimental group specimens. When the aerogel content was 400 mL, the average thermal conductivity was 0.1901 W/m·K. For the specimens with 600 mL (A-600) aerogel content, it was 0.1766 W/m·K, for 800 mL (A-800) aerogel content, it was 0.1720 W/m·K, and for 1000 mL (A-1000) aerogel content, it was 0.1616 W/m·K. It was observed that as the aerogel content increased, the thermal conductivity decreased. During the fabrication process of the specimens made from the pure experimental group, it was noted that aerogel, suspected to have incomplete bonding due to its hydrophobicity, was expelled along with moisture. The thermal diffusivity varied between 0.239 and 0.1948 mm^2^/s depending on the aerogel content.

**Figure 8:**
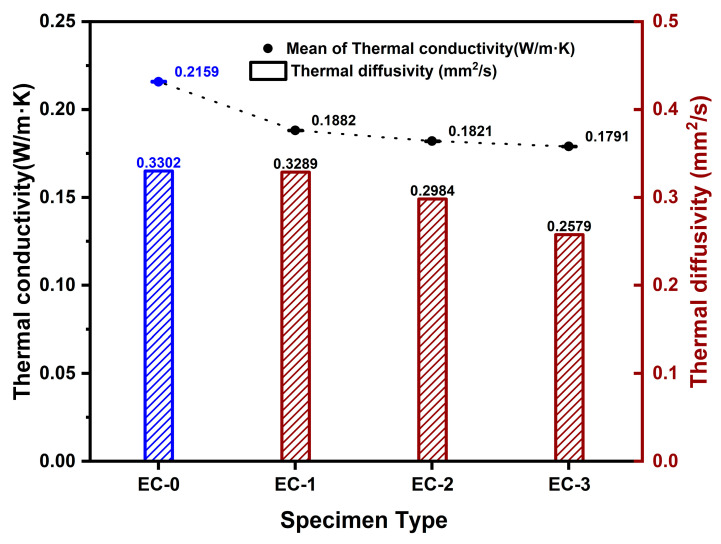
Variation of thermal conductivity and thermal diffusivity with aerogel content in composite-based materials. (Blue: composite control group, Red: composite experimental group).

The thermal conductivity of the composite control group specimen, mixed with 30 wt% ceramic binder and 40 wt% expanded graphite, along with aerogel, was determined to be 0.2157 W/m·K. When the aerogel content was increased to 200 mL, 600 mL, and 1000 mL in the composite control group specimens, the thermal conductivity ranged from 0.1883 to 0.1791 W/m·K. In the composite control group specimens where aerogel was mixed under the conditions of ceramic binder and expanded graphite, the thermal conductivity varied with the aerogel content: 0.1882 W/m·K for 200 mL, 0.1821 W/m·K for 600 mL, and 0.1791 W/m·K for 1000 mL. A decreasing trend in thermal diffusivity, ranging from 0.3289 to 0.2579 mm^2^/s, was observed with increasing aerogel content. Figure 8 illustrates the individual thermal conductivity and thermal diffusivity of the composite control and composite experimental group specimens.

## 4. Discussion

Thermal conductivity is an intrinsic property of a material that indicates its ability to transfer heat. It is typically measured in W/m⋅K and denoted by symbols such as *k*, λ, or κ. For instance, at 1 atmosphere and 293 K (20 °C), the thermal conductivity of air is approximately 0.025 W/m⋅K, while that of water is around 0.5918 W/m⋅K. Formula (2) is commonly used as the basic formula for thermal conductivity, with the most significant factor being the heat transfer quantity, denoted as Q. As this value increases, indicating better heat transfer, the thermal conductivity of the material increases accordingly. A higher *K* value signifies greater heat loss, as materials with higher thermal conductivity allow more heat to pass through.

(3) $$K=\frac{Q*l}{A*∆T}$$

where *K* = thermal conductivity (W/(m·K)), *Q* = heat transfer (W), *A* = heat transfer area (m^2^), and Δ*T* = temperature difference (K or °C)

According to a study by Cha et al., the thermal conductivities of conventional building materials measured using the Heat Flow Meter 436 (HFM 436) were found to be 0.1254 W/m·K for reinforced flooring, 0.2021 W/m·K for fire-resistant gypsum board, and 0.0415 W/m·K for polystyrene [24]. Through our research, the composite specimens based on recycled paper and aerogel showed the best thermal conductivity of 0.1616 W/m·K when the aerogel content was 1000 mL. This value is comparable to those suitable for flooring materials such as laminate flooring and gypsum board.

The *R*-value (thermal resistance) is a commonly used metric for evaluating the performance of insulation materials, expressed in units of m^2^·K/W. Formula (3) expresses the method for calculating the *R*-value. Table 6 presents the *R*-value values of the experimental specimens.

(4) $$R=\frac{L}{k}$$

where *R* = thermal resistance (m^2^·K/W), *L* = thickness (M), and *k* = thermal conductivity (W/(m·K)).

**Figure 9:**
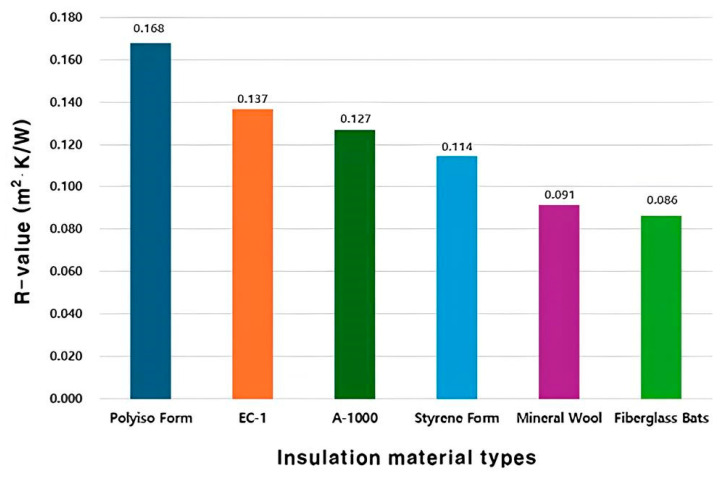
*R*-value by insulation material types.

According to a study by Acharya et al., the *R*-value of architectural materials composed of aerogel was reported to be 0.26 m^2^·K/W. Figure 9 shows the *R*-value values of conventional architectural insulation materials and the mixed insulation materials used in this study [25]. For the pure experimental group specimens with recycled paper and 1000 mL aerogel added, the *R*-value was confirmed to be 0.127 m^2^·K/W. Furthermore, in the case of the composite experimental group specimens under the mixing conditions of ceramic binder and expanded graphite, the specimen with 200 mL aerogel showed the highest *R*-value of 0.137 m^2^·K/W.

## 5. Conclusions

Firstly, adding aerogel, a porous material, to recycled paper to enhance thermal insulation performance resulted in the highest value of 0.1616 W/m·K at the highest concentration of 1000 mL. This value indicates a 16.66% improvement in thermal insulation performance compared to the control group. The architectural material incorporating 1000 mL aerogel into recycled paper showed values suitable for use as flooring materials and gypsum boards in conventional architectural materials.

Secondly, the thermal conductivity value of the composite control group was confirmed to be 0.2157 W/m·K. Additionally, among the composite control group specimens, the specimen with 1000 mL of added aerogel showed a thermal conductivity value of 0.1791 W/m·K. The reduced value demonstrates a 17.06% improvement in thermal insulation performance compared to the control group.

Lastly, in terms of *R*-value, the specimen mixed with 1000 mL aerogel in the composite experimental group showed an *R*-value of 0.137 m^2^⋅K/W. The *R*-value values of EC-1 and A-1000 were lower than those of Styrene Foam.

In this study, consistent with prior aerogel research, no significant increase in thermal conductivity was observed. This can be attributed to the chosen specimen preparation method involving compression, resulting in high specimen density. It is speculated that this high density impedes the formation of air gaps crucial for thermal insulation. Nevertheless, this study quantitatively confirms the potential for further development and commercialization of cellulose-based porous aerogel composite building materials through additional research.

## Figures and Tables

**Table 1:** Physical properties of powdered silica aerogel.

Color	White, Translucent
Density (kg/m^3^)	<40
Thermal conductivity (W/mK at 25 °C)	0.011
Porosity (%)	99
Aperture (nm)	10~50
Pore volume (cm^2^/g)	3.0~3.6
Particle size (μm)	1~10
Specific surface area (m^2^/g)	650~1250
Speed of sound (m/s)	~100

**Table 2:** Material-specific compositions of the pure experimental group specimens.

Test Specimens Category	Paper (g)	Aerogel (mL)	Organic Compounds (g)
A-400	150	400	600
A-600	150	600	600
A-800	150	800	600
A-1000	150	1000	600

**Table 3:** Material-specific compositions of the composite experimental group specimens.

Test Specimens Category	Paper (g)	Aerogel (mL)	Organic Compound (g)	Ceramic Binder (wt%)	Expandable Graphite (wt%)
EC-1	150	200	600	30	40
EC-2	150	600			
EC-3	150	1000			

**Table 4:** Physical properties of the experimental specimen.

Test Specimen Category	Weight before Drying (g)	Weight after Drying (g)	Height (cm)	Area (cm^2^)	Volume (cm^3^)	Density (g/cm^3^)	Water Content (%)
A-400	221.5	97.6	2.24	100	224	0.44	55.94
A-600	188.6	84.6	2.1	100	210	0.40	55.14
A-800	200.4	86.8	2.13	100	213	0.41	56.69
A-1000	188.6	84.8	2.05	100	205	0.41	55.04
EC-1	232.5	108.4	2.57	100	257	0.42	53.38
EC-2	231	86.1	2.08	100	208	0.41	62.73
EC-3	222.75	92.2	2.18	100	218	0.42	58.61

**Table 5:** EDS analysis results of the test group specimens.

Element	Pure Test Group Specimen		Composite Test Group Specimen	
	Weight%	Atomic%	Weight%	Atomic%
Carbon (C)	43.86	51.56	44.04	51.93
Oxygen (O)	53.25	46.99	52.09	46.12
Silicon (Si)	2.89	1.46	3.87	1.95
Totals	100.00	-	100.00	-

**Table 6:** R-value values by specimens.

A Test Specimens	A-400	A-600	A-800	A-1000	EC-1	EC-2	EC-3
R-value (m^2^⋅K/W)	0.118	0.119	0.124	0.127	0.137	0.114	0.122

## Data Availability

Data are contained within the article.
